# DISubNet: Depthwise Separable Inception Subnetwork for Pig Treatment Classification Using Thermal Data

**DOI:** 10.3390/ani13071184

**Published:** 2023-03-28

**Authors:** Savina Jassica Colaco, Jung Hwan Kim, Alwin Poulose, Suresh Neethirajan, Dong Seog Han

**Affiliations:** 1 School of Electronic and Electrical Engineering, Kyungpook National University, Daegu 41566, Republic of Korea; savinacolaco@knu.ac.kr (S.J.C.); jkim267@knu.ac.kr (J.H.K.); 2 School of Data Science, Indian Institute of Science Education and Research (IISER), Thiruvananthapuram 695551, India; alwinpoulosepalatty@iisertvm.ac.in; 3 Farmworx Wageningen Institute, 6706 JS Wageningen, The Netherlands; sneethir@gmail.com

**Keywords:** animal welfare, depthwise separable layer, image classification, inception, thermal data

## Abstract

**Simple Summary:**

Thermal imaging is gaining popularity in poultry, swine, and dairy animal husbandry for detecting disease and distress. In this study, we present a depthwise separable inception subnetwork (DISubNet) for classifying pig treatments, offering two versions: DISubNetV1 and DISubNetV2. These lightweight models are compared to other deep learning models used for image classification. A forward-looking infrared (FLIR) camera captures thermal data for model training. Experimental results show the proposed models outperform others in classifying pig treatments using thermal images, achieving 99.96–99.98% accuracy with fewer parameters, potentially improving animal welfare and promoting sustainable production.

**Abstract:**

Thermal imaging is increasingly used in poultry, swine, and dairy animal husbandry to detect disease and distress. In intensive pig production systems, early detection of health and welfare issues is crucial for timely intervention. Using thermal imaging for pig treatment classification can improve animal welfare and promote sustainable pig production. In this paper, we present a depthwise separable inception subnetwork (DISubNet), a lightweight model for classifying four pig treatments. Based on the modified model architecture, we propose two DISubNet versions: DISubNetV1 and DISubNetV2. Our proposed models are compared to other deep learning models commonly employed for image classification. The thermal dataset captured by a forward-looking infrared (FLIR) camera is used to train these models. The experimental results demonstrate that the proposed models for thermal images of various pig treatments outperform other models. In addition, both proposed models achieve approximately 99.96–99.98% classification accuracy with fewer parameters.

## 1. Introduction

Over the past few years, the number of applications for image classification has significantly increased. The goal of image classification is to determine the class to which a target object belongs. Classification is required whenever an object is assigned to a specific group or class based on the characteristics associated with that object. Image classification has many applications, including medical image analysis, human and animal face recognition, and monitoring and classifying animal behaviour [1]. It can be difficult to distinguish an object in an image if it is obscured by background clutter, noise, poor image quality, or other factors. Furthermore, the visible spectrum has limitations, such as lighting conditions and shadows, that could be overcome by thermal imaging. Thermal imaging is a non-destructive testing method that can be utilized to determine the surface temperature of objects. Thermal imaging is increasingly utilized in animal welfare to increase farm production efficiency. Calves [2], poultry [3], and pig production [4] have been evaluated for animal welfare using thermal imaging. In addition, it is used to identify the temperature increase in pigs to predict their health [5].

In computer vision, animal classification using thermal images has been a crucial field of study. Continuous automatic systems for animal welfare typically provide information by collecting raw data and identifying key features through deep learning techniques. Farmers were better able to understand specific animal needs, such as welfare [6,7,8], and reproductive efficiency [9,10], with the aid of this method. The problem with automatic systems is that they use all nearby natural objects to represent animals in a scene, rather than just the animals themselves. In addition, animals can be viewed from various perspectives, scales, and shapes, as well as under different lighting conditions. However, this could be resolved using thermal images for animal classification. Thermal images capture heat emitted by animals, and these data can be used to identify patterns and detect abnormalities that are not visible to the naked eye. The use of thermal imaging in livestock applications has the potential to improve animal welfare, increase productivity, and reduce the environmental impact of livestock production. Ongoing research and development in this field will likely result in even more advanced applications in the near future. Thermal imaging can be used in livestock to detect indications of illness or injury. Changes in body temperature, for example, can suggest the existence of a fever, which is a typical sign of many disorders [11]. Thermal imaging can be used to detect estrus, which indicate when a female animal is in heat [12]. Furthermore, this information can be used to improve breeding programs and increase reproductive efficiency. Stress detection in livestock using elevated body temperature or changes in respiratory patterns for animal welfare [13]. Thermal imaging can be used to monitor individual animal growth as well as environmental factors such as temperature and humidity in livestock facilities [14]. Since there are many different animal classes, each with a complex intra-class variability and inter-class similarity, methods for classifying human faces have been developed with high accuracy, but those methods are incredibly inaccurate for classifying animal faces [6]. With each approach having advantages and disadvantages, researchers have tried various approaches to address these issues.

Convolutional neural network (CNN)-based classification techniques have, however, drawn a lot of attention in recent years. Deep learning methods involve representation learning and have multiple levels of representation [1]. Each of the modules that make up these algorithms transforms the representation at one level into a representation at a higher, more fundamental level while still being relatively simple but non-linear. As a result, a combination of these transformations can be used to learn quite complex functions. The higher-level representation enlarges aspects of data input that are important for distinguishing and suppressing irrelevant changes in animal classification tasks. The benefits of deep learning techniques have been successfully demonstrated in numerous applications where the input values are characterized by high dimensionality, enormous quantities, and highly structured data [15]. Deep learning techniques have a good performance and are, therefore, widely used in animal classification. Additionally, they have been widely used with thermal images [16]. Deep learning tools are incredibly helpful in image classification because the structure of the image is made up of millions of pixels that can be aligned into distinct objects [17]. The development of deep learning models has practical implications for pig farm management, allowing farmers to make data-driven decisions that improve pig health, welfare, and productivity. The advancement of neural models has greatly enhanced our ability to predict and manage various aspects of pig farming. Deep learning can help farmers optimize feeding programs and predict growth rates by analyzing large datasets of pig growth and feed intake [18]. Deep learning techniques can help predict disease outbreaks early by finding patterns in pig behavior and health data. These data can be utilized to develop early warning systems and guide disease management strategies [19]. They may be used to predict temperature and humidity levels in pig barns, which can assist farmers in maintaining optimal environmental conditions for pig growth and health [20]. Deep learning models can enable farmers to identify breeding pairs that are likely to generate high-quality offspring with desirable traits by examining large datasets of genetic and phenotypic data [21].

To achieve greater accuracy, the general trend has been to create deeper and more complex networks [22]. These improvements in accuracy are necessarily making networks less effective in terms of size and speed. The recognition tasks in many real-world applications, including robotics, self-driving cars, and augmented reality, must be completed promptly on a platform with constrained computational resources [23]. To solve this issue, scaling CNN can improve accuracy while keeping the model lightweight and efficient. We propose a lightweight model that employs depthwise convolution layers and inception modules to reduce computational load while increasing accuracy with fewer parameters. We use thermal images instead of standard RGB images to overcome varying lighting and background conditions.

The main contributions of the paper are as follows:

1. We propose a depthwise separable inception subnetwork (DISubNet), a lightweight model for pig treatment classifications that consist of depthwise separable layers and an inception module.
2. We propose two versions of DISubNet: DISubNetV1 and DISubNetV2. The models are modified based on the concatenation of depthwise layers and inception modules.
3. Experiments are carried out on the pig image thermal dataset collected from the FLIR camera. The collected dataset consists of four pig treatment categories, such as isolation after feeding (IAF), isolation before feeding (IBF), paired after feeding (PAF), and paired before feeding (PBF).
4. Detailed experiments are conducted on both versions of DISubNet models with other image classification models using various evaluation metrics.

The rest of the paper is organized as follows: Section 2 provides the related works on image classification. The proposed models are explained in detail in Section 3. Section 4 provides details about the experiment. Section 5 contains the results of the experiments and their discussion. Finally, we conclude in Section 6.

## 2. Related Work

### 2.1. Image Classification Methods

Deep learning methods are commonly used in image classification tasks. The image classification process begins with the input image and ends with a classified result based on the class. The same principle applies to animal classification. The CNN-based animal classification system can be divided into three phases: pre-processing, feature learning, and classification. Firstly, to maximize the impact of factors that influence the animal classification algorithm, the input image undergoes a rescaling and image augmentation process in the pre-processing stage [24]. Second, in the feature learning step, the convolution algorithm is used to calculate the features of the input image. Finally, in the classification step, a predictive model is constructed using the features from the training data [25]. These predictive models estimate their class labels by comparing learned features from training data with test data or validation data [26]. The output classes are specific, and the user can discover the precise name of the class based on the prediction ratio. Animal image classification has previously been carried out using a variety of conventional classifiers, including support vector machine (SVM) [27,28] random forest (RF) [29,30], and decision tree (DT) [31,32,33]. In various settings, the use of an ensemble has grown in popularity. An ensemble is a supervised learning strategy that uses multiple models to boost the performance of a single model [7]. Recent research has mainly used deep learning techniques due to the promising results it has demonstrated in challenging computer vision tasks. In their work on animal species identification, Villa et al. [34] used the AlexNet [35], VGGNet [36], GoogLeNet [37], and ResNets [23] to analyze images of animals taken with a digital camera and an infrared sensor. The wildlife detector [38] was provided as a CNN model that trains a multi-class classifier while also learning a binary classification with two classes: animal and non-animal. There are a few popular methods to divide and categorize animals in camera-trap images [39]. Animal recognition methods such as robust layer principal component analysis for segmentation, CNN for feature extraction, the least absolute shrinkage and selection operator (LASSO) for characteristics, and the SVM for classification of mammalian genera have been used in the Colombian forest [40]. As a classification model, ResNet50, ResNet101, ResNet152, GoogLeNet, and MixtureNet, which are all frequently used CNN models, were utilized [40]. CNNs have great potential in agriculture and livestock contexts for improving animal health and welfare, as well as for increasing efficiency and productivity on farms. As machine learning and computer vision technologies continue to advance, we can expect to see more innovative applications of CNN in the agricultural industry. CNNs can be trained to recognize individual animals, such as pigs or cows, based on their facial features or body markings [41]. This can be useful for tracking animal health and growth over time. CNNs can also be used to analyze animal behavior, such as monitoring pig or cow facial expressions to detect signs of pain or distress [42]. Tools such as ChickTrack use CNNs to track chicken activity levels, which can help farmers to monitor the health and welfare of their birds [43]. CNNs can help to automatically record and manage animals using different sensor technologies [44].

### 2.2. Model Design and Efficiency

For the past few years, researchers have been working on fine-tuning deep neural architectures to achieve the best possible balance between accuracy and performance. Small and effective neural networks are becoming increasingly popular in animal welfare [45,46]. Both compressing pre-trained networks and training small networks directly fall under the broad categories of many different approaches. There have been significant advancements over early designs such as AlexNet, VGGNet, GoogLeNet, and ResNet thanks to both manual architecture search and training algorithm improvements. In recent years, there has been significant progress in algorithmic architecture exploration, including hyperparameter optimization [47] network pruning [48] and connectivity learning [49]. As seen in ShuffleNet [50] or the addition of sparsity, much work has also gone into changing the connectivity structure of the internal convolutional blocks. Another advantage of deep learning is creating distributed representations that generalise newly learned characteristics and those observed during training. As a result, each of these representations can help model similar representations in other domains [49]. However, it is important to note that deep learning models are frequently complex models that involve the use of a large number of computational resources. Therefore, the goal of this paper is to design the model structure for the classification of pig treatments using thermal images with a focus on the need for smaller and more effective models.

## 3. Materials and Methods

In this section, we describe the various models used in the experiments, including LeNet5 [51], AlexNet, VGGNet, Xception [52], CNN-LeakyReLU [53], CNN-inception, and the proposed DISubNet model. These models are compared for the classification of the pig treatments.

### 3.1. Image Classifcation Models

One of the first pre-trained models is LeNet5, which recognises handwritten and machine-printed characters. The main reason that the model is popular is due to its straightforward structure. It is an image classification multi-layer convolution neural network which is made up of five layers that have learnable parameters. This network comprises three sets of convolutional layers, followed by a combination of average pooling layers and two fully connected hidden layers [51]. The images are classified using a softmax classifier. AlexNet won the Imagenet large-scale visual recognition challenge in 2012. The network depth in this model was increased when compared to the LeNet5 network. It has eight layers with learnable parameters. The model has five layers, the first of which is a max-pooling combination, followed by three fully connected layers [35]. The layers use rectified linear unit activation (ReLU) as their activation function, which speeds up the training process. Dropout layers are also used in the model to avoid overfitting. The final layer employs softmax as its activation function. So, as we progress deeper into the architecture, the number of filters grows. As a result, it extracts more features as we progress deeper into the architecture. Furthermore, the filter size is decreasing, indicating that the initial filter was larger and that as we progress, the filter size is decreasing, resulting in a decrease in the feature map shape. The University of Oxford’s visual geometry group (VGGNet) [36] created a deep convolutional neural network, which is widely used in computer vision fields. It comprises VGG-16 or VGG-19, which refer to the 16 and 19 convolutional layers, respectively. Xception employs depthwise separable convolutions [52]. It was developed by researchers at Google. They interpreted inception modules in CNN as an intermediate step between conventional convolution and the depthwise separable convolution in which a depthwise convolution is followed by a pointwise convolution.

### 3.2. Modified CNN Models

**Figure 1:**
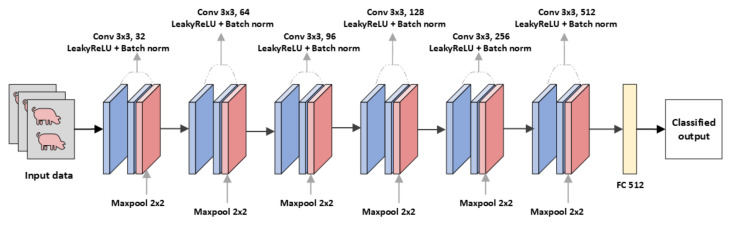
CNN-LeakyReLU: Convolution neural network with LeakyReLU and batch normalization.

The CNN model with LeakyReLU [53] is a straightforward sequential model consisting of several convolutional layers and a batch normalization layer. Following the convolutional layers is LeakyReLU, which is based on ReLU but has a small slope for negative values rather than a flat slope. To reduce the spatial dimension of the feature map, max pooling is applied after each even convolution layer. The convolution layer has a filter size of 3×3 and a pooling size of 2×2 across all layers. Figure 1 depicts the CNN-LeakyReLU model structure.

**Figure 2:**
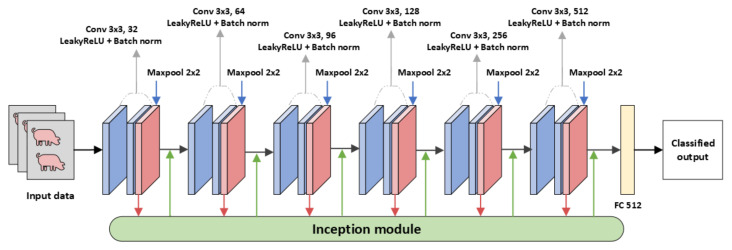
CNN-Inception: Convolution neural network with Inception module.

**Figure 3:**
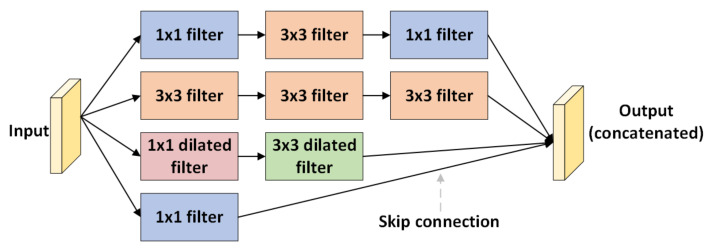
Inception module with different filters for extraction.

Similar to the CNN-leakyReLU model structure, the model consists of convolutional layers and batch normalization layers. The max pooling is followed after every even convolution layer. Convolutional layers are made up of 3×3 filters in each layer. After every two convolution layers, max pooling with a 2×2 filter is applied to reduce the spatial dimension of the feature map. Figure 2 shows a representation of CNN-inception. To extract features, the model is further modified with a tunable inception module [37] consisting of filters such as 1×1, 3×3, and dilation filters. Dilated filters increase the area covered by the input image without pooling. The goal is to extract more information from each convolution operation’s output. The different feature extraction from filters aids in focusing on different parts of images to detect complex patterns. In addition, the inception module includes a skip connection for identity mapping. The class scores will be processed by the fully connected layer, resulting in a volume in size, where each of the four numbers corresponds to a class score. The filters used in the inception module are more specifically shown in Figure 3.

### 3.3. Proposed Model for Pig Treatment Classification

**Figure 4:**
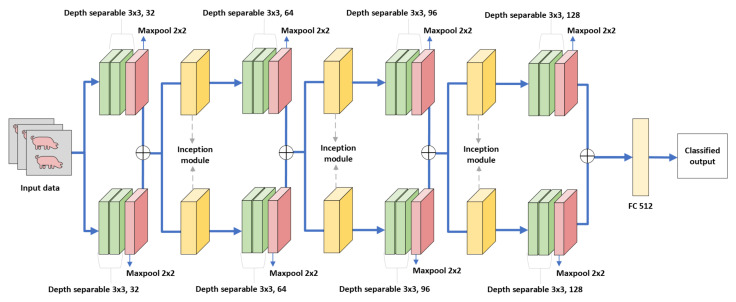
DISubNetV1: Depthwise separable convolution with inception module subnetwork. The output from depthwise layers is concatenated in this model and fed into the inception module for further extraction.

**Figure 5:**
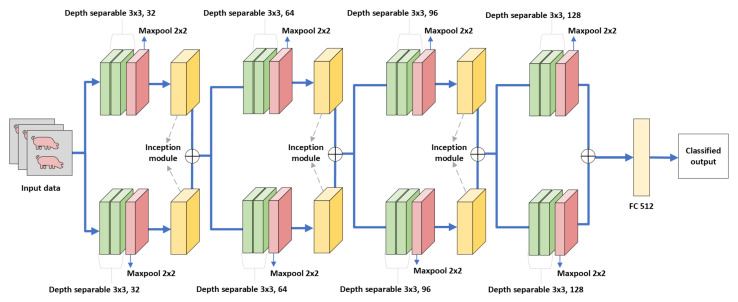
DISubNetV2: Depthwise separable convolution with inception module subnetwork. In this model, the inception modules are concatenated and fed as input to subsequent layers.

In comparison to large convolutional neural networks such as LeNet5, AlexNet, and VGGNet, DISubNet aims to make all of these networks smaller with fewer parameters while maintaining the same level of accuracy or even improving model generalization using fewer parameters. Larger networks are more prone to overfitting and raise the computation complexity. CNNs can also benefit from the extraction of features at different scales. Therefore, we propose DISubNet comprising of two subnetworks with alternating depthwise separable convolution layers [54] and an inception module. Additionally, we propose two DISubNet versions, DISubNetV1 and DISubNetV2. Figure 4 and Figure 5 provide detailed information about the DISubNetV1 and DISubNetV2 models.

The depthwise separable convolution layers from both subnetworks are concatenated in the DISubNetV1. The concatenated output from both subnetworks is fed into the inception module. In the DISubNetV2 model, we concatenate inception modules from both subnetworks and feed them as input to the depthwise layers.

**Figure 6:**
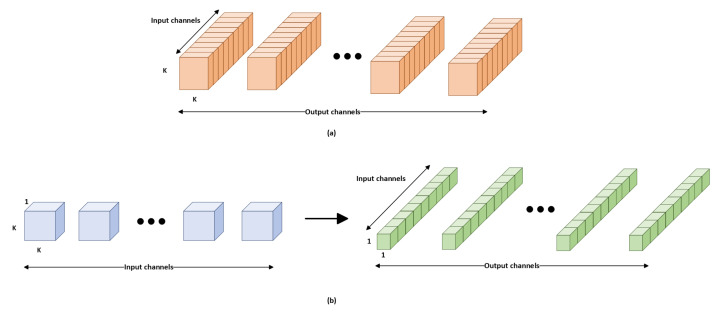
Comparison of depthwise separable and standard convolution layers. K refers to the kernel size. (**a**) Standard convolution filter, (**b**) Deptwise convolution followed by pointwise convolution.

Depthwise separable convolutions, also known as separable convolutions, are one approach. It separates the channel and spatial convolutions normally combined in convolutional layers. The number of output channels equals the number of input channels because we apply one convolutional filter to each output channel. We then apply a pointwise convolutional layer after the depthwise convolutional layer. A pointwise convolutional layer is a convolutional layer with a 1×1 kernel. A 1×1 kernel is to use non-linearity. A ReLU activation function is applied after each layer of a neural network. The inception module follows the same structure as the CNN-inception model. The inception modules from both subnetworks are concatenated and become inputs to the subsequent layers. Figure 6 illustrates a comparison of depthwise convolution layers and standard convolution layers.

The DISubNet models can regularize our model by reducing the number of parameters and the number of computations required during training or inference. Additionally, the model takes advantage of the inception module’s capacity to extract features from input data at different scales by employing different convolutional filter sizes. DISubNet models use computing resources efficiently with minimal increase in computation load.

## 4. Experimental Setup

### 4.1. Dataset

**Figure 7:**
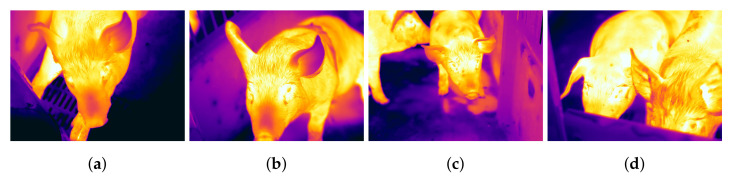
Thermal images of different pig treatments [53]. (**a**) IAF, (**b**) IBF, (**c**) PAF, and (**d**) PBF.

The data were collected by Wageningen University and Research using a FLIR camera. The FLIR T1020 with a standard 28-degree lens and FLIR Thermal Studio was used to acquire the thermal videos. Thermal videos of different pig treatments are included in the dataset. For simplicity, we extract the images from the video and convert them to grayscale with 62,800 images in total. The pigs were filmed in pairs and separated before and after feeding as shown in Figure 7, resulting in four treatment groups: isolation after feeding (IAF), isolation before feeding (IBF), paired after feeding (PAF), and paired before feeding (PBF).

The pigs were classified into four treatment groups to assess animal welfare during physical separation and transport using a thermal camera. These labels represent the four different pig treatments as well as the experiment’s required classified output. The thermal images of the IAF and IBF contained single pigs. The images in the PAF and PBF contain multiple pigs. Arousal in pigs is manipulated by delayed feeding due to short-term food restriction. Delaying feeding often increases the rate of eating, indicating higher arousal. Restrictive feeding tends to enhance aggression in pigs, which may result in adversarial social behavior when dealing with other pigs in the pen. To be able to build solutions and animal welfare monitoring systems for overcoming aggression and tail biting, it is crucial to analyze the impact of feeding intervals and pen mate manipulation behavior. The abnormal behavior of the pigs may be related to the redirection of the pig’s exploratory behavior, such as the ability to engage with the pen mate whether maintained in groups or in isolation. Hence these four treatments namely IAF, IBF, PAF, and PBF were chosen to understand the effect of feeding intervals and access to socializing conditions on the behaviour of pigs. The entire dataset is divided into 60, 20, and 20 ratios for train, test, and validation data, respectively. As a result, the training data have 37,680 images, and the test data have 25,120 thermal images.

### 4.2. Implementation Details

The experiment uses images resized to 112×112 resolution. The models were trained using the Keras framework with a batch size of 32 and epochs of 100. All models have been trained on the Nvidia GeForce RTX 2070 SUPER GPU. For network training, the Adam optimization [55] method is used, which is an effective stochastic optimization that only requires first-order gradients and needs less memory. It combines the benefits of two common methods: AdaGrad [56], which works well with sparse gradients, and RMSProp [57], which works well in non-stationary and online settings. Instead of stochastic gradient descent, Adam is used to iteratively update network weights based on training data. The Adam technique is used to optimize the model at various learning rates, such as $10^{-2}$, $10^{-3}$, and $10^{-4}$.

### 4.3. Loss Function

The categorical cross-entropy loss is also called softmax loss. It is closely related to the softmax function because categorical cross-entropy loss almost exclusively affects networks with a softmax layer at the output. The categorical cross-entropy loss is only employed in multi-class classification tasks where each sample precisely belongs to one of the *C* classes. Each sample is given a ground truth label, an integer value between 0 and C−1. A one-hot encoded vector of size *C* with a value for the correct class and zeroes everywhere can represent the label. The cross-entropy algorithm takes two discrete probability distributions as input and produces a single real-valued number indicating the correlation of both probability distributions. The categorical cross-entropy loss function is represented as,

(1) $$E_{\mathrm{loss}}\left(y,s\right)=-\sum_{i=1}^{C}y_{i}\log(s_{i})$$

where *C* denotes the number of distinct classes and *i* denotes the *i*-th element of the vector. The one-hot encoded label is fed into *y*, and the probabilities generated by the softmax layer are placed in *s*. The lower the cross-entropy, the closer the two probability distributions are to one another.

### 4.4. Activation Function

ReLU is a non-linear activation function with output zero if the input *x* is less than zero and output equivalent to the input if the input is greater than zero. Hence, the ReLU function takes the maximum value of *x*. It has more advantages than the sigmoid function, which has more backpropagation errors. ReLU could be represented as

(2) f(x)=max(x,0)

However, there are a few drawbacks to ReLU, including the fact that it is not zero-centred and is not differentiable at zero. Another issue that the ReLU faces is the dying ReLU problem in which some ReLU neurons essentially die for all inputs and remain inactive regardless of input, resulting in no gradient flow and affecting performance. As a result, we use LeakyReLU in experiments where there is a small negative slope so that instead of not firing at all for large gradients, the neurons do output some value, making the layer much more optimized. LeakyReLU is represented as

(3) f(x)=max(0.1x,x)

### 4.5. Evaluation Metrics

The accuracy, loss, F1 score, precision, recall, and number of parameters are used to compare the various models. The accuracy of the validation data measures how often the classifier predicts correctly. The precision metric explains how many of the correctly predicted cases were positive. It is useful in situations where false positives are more serious than false negatives. Recall describes how many of the actual positive cases the model correctly predicted. It is useful when false negatives are more concerning than false positives. The F1 score is derived from precision and recall metrics. It is also used to balance precision and recall when dealing with uneven dataset distribution. The evaluation metrics for the model are described as

(4) $$\mathrm{Accuracy}=\frac{\mathrm{TP}+\mathrm{TN}}{\mathrm{TP}+\mathrm{TN}+\mathrm{FP}+\mathrm{FN}}$$

(5) $$\mathrm{Precision}=\frac{\mathrm{TP}}{\mathrm{TP}+\mathrm{FP}}$$

(6) $$\mathrm{Recall}=\frac{\mathrm{TP}}{\mathrm{TP}+\mathrm{FN}}$$

(7) $$F1_{\mathrm{score}}=\frac{2\times \mathrm{Recall}\times \mathrm{Precision}}{\mathrm{Recall}+\mathrm{Precision}}$$

where TP, TN, FP, and FN represent true positive, true negative, false positive, and false negative, respectively. The confusion matrix is a popular performance metric for classification problems with two or more classes as output.

## 5. Results and Discussion

### 5.1. Model Comparison

**Figure 8:**
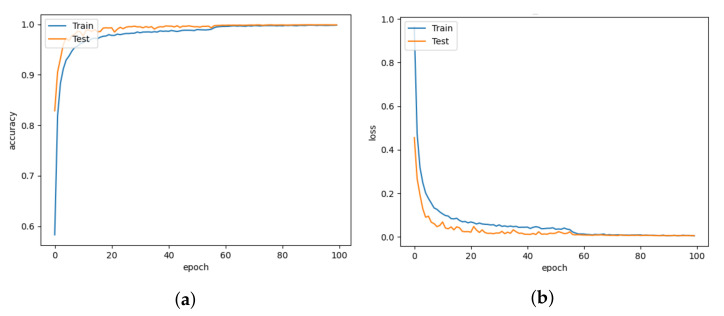
LeNet5 for learning rate = 0.001. (**a**) Model accuracy. (**b**) Model loss.

We evaluated and visualized our results using an accuracy, loss, and confusion matrix. For our experiment, we have modified the LeNet5 for input data of 112×112. The network consists of two sets of convolution layers followed by max pooling. The filter size for the convolution layer is 5×5 with stride 1, and the pooling size is 2×2. There are 500 neurons in the hidden layers. The activation function used in this model is the ReLU activation function. With 19.6 M parameters, LeNet5 has an accuracy of 99.9%. After a certain epoch, the model converges but slightly overfits the model. With a learning rate of $10^{-3}$, the LeNet5 was able to close the generalization gap with a 0.006 error. LeNet5 is limited by the availability of computing resources because processing higher-resolution images require larger and more convolutional layers, which are difficult to implement. Figure 8a,b show the accuracy and loss plot of the LeNet5 with slight overfitting at the beginning of the training.

**Figure 9:**
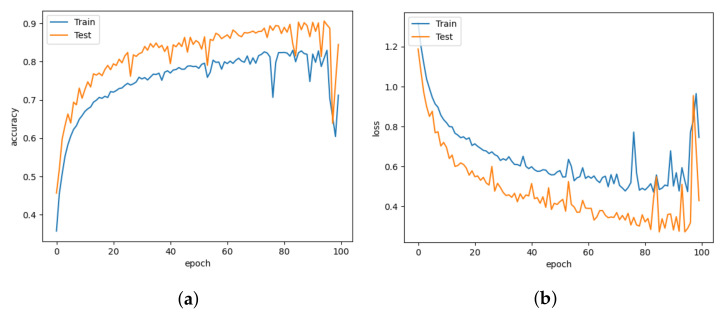
AlexNet for learning rate = 0.001. (**a**) Model accuracy. (**b**) Model loss.

The AlexNet model is slightly modified to use 4 convolutional layers instead of 5 for a 112×112 input size. The convolutional layers employ 11×11, 5×5, and 3×3 filter sizes. As a result of the varying convolution filter sizes, the network can learn various spatial patterns at different scales. The max pooling is applied with the size of 3×3 with stride 2. Despite having an accuracy of 90.22% with many parameters, AlexNet has several misclassified images. In comparison to LeNet5, AlexNet has 23.3 M parameters because of the addition of layers. As a result, AlexNet is not only a large model but also highly prone to overfitting. With a 0.27 error value, AlexNet has more errors than LeNet5. Figure 9a,b shows that AlexNet shows an accuracy plot and a loss plot.

**Figure 10:**
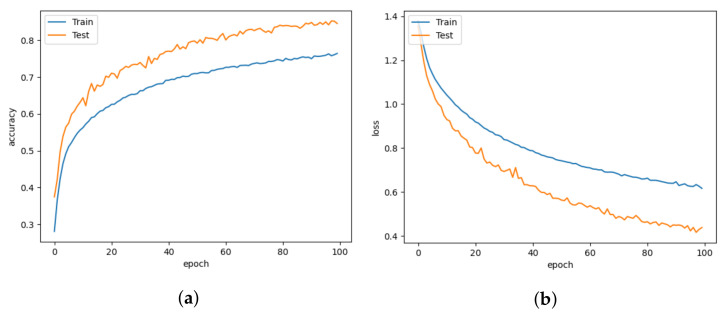
VGGNet for learning rate = 0.001. (**a**) Model accuracy. (**b**) Model loss.

In this paper, we compare the 16-layer VGG-16 model with other models. VGG-19 was excluded from the experiment because it has a 55 M number of parameters. The convolutional layers are followed by single max pool layers. The layers use a 3×3 kernel size for a minimal receptive field. These are followed by the ReLU unit, which reduces training time compared to AlexNet. The number of depth layers has increased, and the hyperparameter tuning process has been simplified using only 3×3 filters. Consequently, increasing the depth of the model structure could enhance generalizability. Additionally, a larger receptive field might be offered. The number of parameters might be decreased by using a smaller filter size. Due to a large convergence gap between train and test data, VGGNet performed worse than other models. Figure 10a,b show that the VGGNet has a smoother learning curve than AlexNet. The model had an accuracy of 85.43% with 17 M parameters. Since the data are not evenly distributed, the VGGNet overfits similarly to AlexNet. With a 0.416 error, the VGGNet has a higher loss value than the AlexNet.

**Figure 11:**
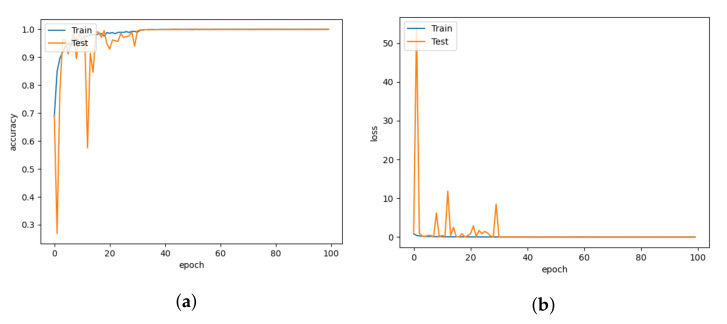
Xception for learning rate = 0.001. (**a**) Model accuracy. (**b**) Model loss.

The Xception model emphasizes the inception hypothesis. Hence, this model is known as the Xception model. Xception provides an architecture that consists of depthwise separable convolution blocks and maxpooling, all of which are connected using shortcuts similar to ResNet implementations. The distinguishing characteristic of Xception is that the depthwise Convolution is not followed by the pointwise convolution; instead, the sequence is inverted. The 1×1 convolutions capture the correlations between channels. Regular 3×3 or 5×5 convolutions capture the spatial correlations within each channel. Hence, 1×1 is applied to each channel, followed by 3×3 to each output. It is similar to substituting depthwise separable convolutions for the inception module. Xception model has the accuracy of 99.95% with 20 M parameters. According to the accuracy and loss plots of the Xception model presented in Figure 11a,b, depthwise separable convolutions reduce overfitting compared to AlexNet and VGGNet. The Xception model has classification accuracy similar to DISubNet V1 and V2 but requires more parameters and a larger model size.

**Figure 12:**
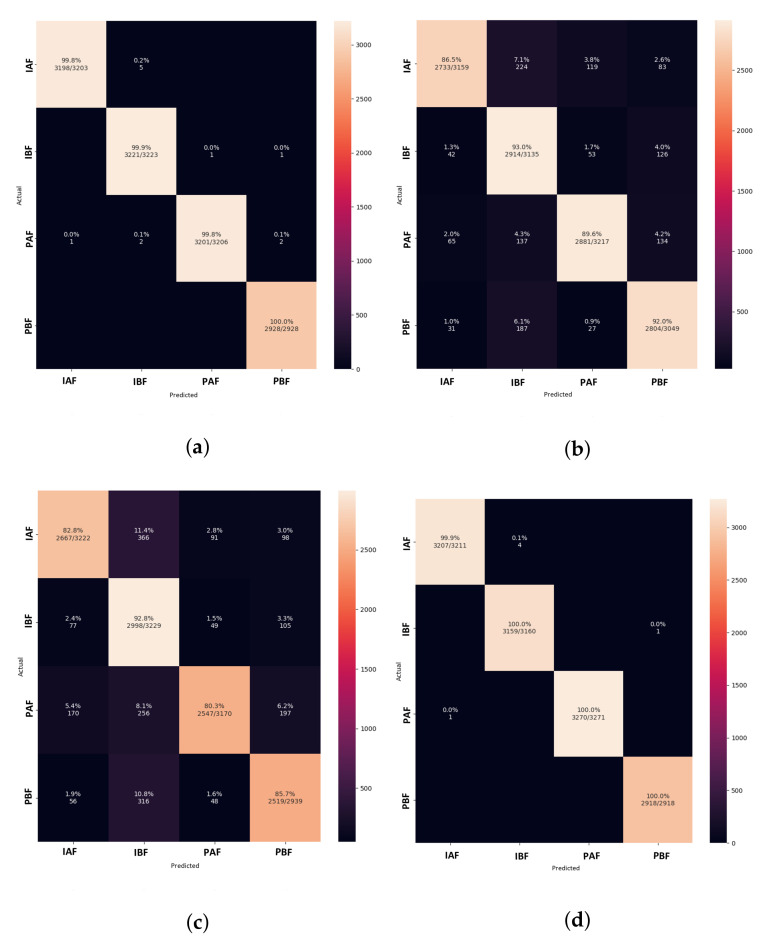
Confusion matrix of image classification models for learning rate = 0.001. (**a**) LeNet5. (**b**) AlexNet. (**c**) VGGNet. (**d**) Xception.

The confusion matrix in Figure 12 shows that the LeNet5 model classifies the paired before feeding treatment class more accurately than the other classes. When compared to other classes, the AlexNet model performs best at classifying isolation before feeding, followed by the class paired before feeding. Among the image classification methods, the VGGNet model illustrates the highly misclassified pig treatments. Furthermore, Xception performs a more accurate classification of pig treatments than LeNet5.

**Figure 13:**
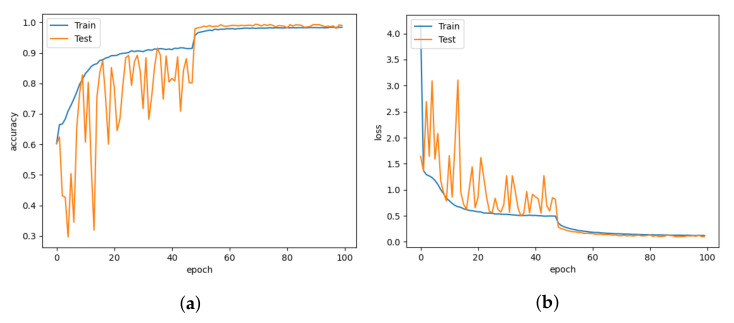
CNN-LeakyReLU for learning rate = 0.001. (**a**) Model accuracy. (**b**) Model loss.

**Figure 14:**
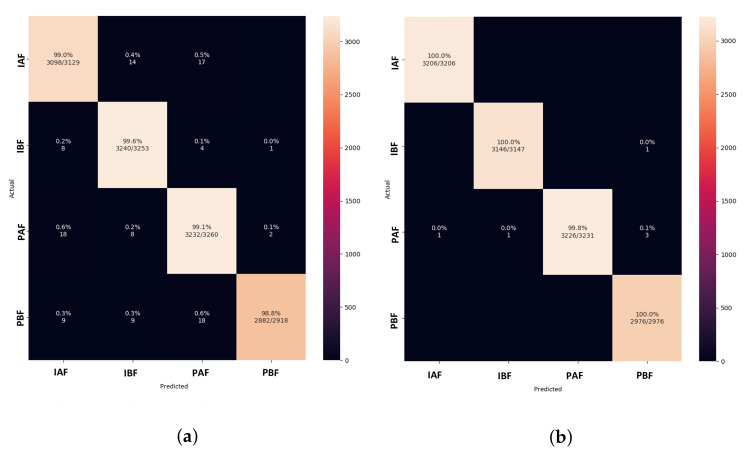
Confusion matrix of modified CNN models for learning rate = 0.001. (**a**) CNN-LeakyReLU. (**b**) CNN-Inception.

In comparison to Lenet5, which uses 19.6 M parameters, the CNN-leakyReLU achieves an accuracy of 99.14% with 7.2 M parameters. Figure 13a,b demonstrate CNN-leakyReLU with more fluctuations in the learning curve at the beginning of the training. The model fluctuated during training due to the uneven data distribution, but it converged successfully after a certain number of epochs. With a 0.097 error, it displays a higher loss value than LeNet5. An L2 regularizer is used to lessen the overfitting of the proposed model. The confusion matrix shown in Figure 14a indicates that most pig treatment classes were also categorized with higher performance.

**Figure 15:**
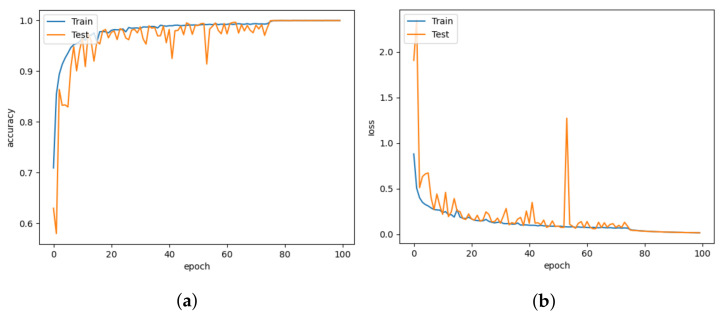
CNN-Inception for learning rate = 0.001. (**a**) Model accuracy. (**b**) Model loss.

The CNN-inception model makes use of the ability of the inception module to focus on different parts of images to find patterns that can be associated with classification labels. Working with different filters to capture the level of abstraction is possible with the inception. As a result, they are not limited to using a single filter size in a single image block, which is then concatenated and passed onto the next layer. After each max pooling, the inception module is added. When the dataset is trained with the CNN-Inception model, it captures better patterns. It thus achieves 99.97% accuracy with a slightly higher number of parameters (i.e., 7.4 M) than CNN-LeakyReLU. Figure 15a,b demonstrate that the CNN-inception model has a better learning and convergence curve than the other models.

In the model, the filters are slid over the entire image, and the dot product of the image and filter values are calculated. The number of filters produces the same number of feature maps as the number of filters, which becomes the parameter for the model to be learned. Deep neural networks that are highly efficient must be large. A neural network had to have several more layers and units within these layers to be considered large. Multi-scale convolutional layers may also be able to learn more. However, large networks are prone to overfitting, and chaining multiple convolutional operations together raises the computational cost of the network [51]. In this case, the inception module is more advantageous. When compared to CNN-LeakyReLU, the model achieves a lower loss of 0.017. As a result, for use in any application, a trade-off between the number of parameters and accuracy could be considered. The CNN-Inception model correctly classifies three treatment categories, as shown by the confusion matrix in Figure 14b.

**Figure 16:**
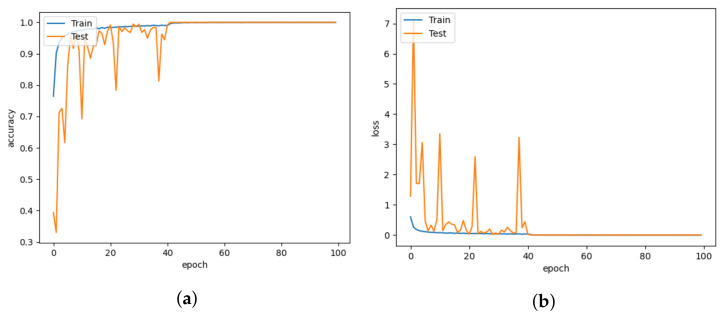
DISubNetV1 for learning rate = 0.001. (**a**) Model accuracy. (**b**) Model loss.

**Figure 17:**
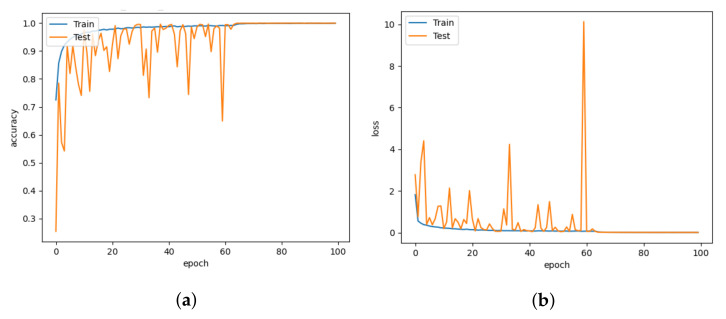
DISubNetV2 for learning rate = 0.001. (**a**) Model accuracy. (**b**) Model loss.

The DISubNet model, which employs depthwise separable convolution layers, has significantly fewer parameters and a slightly lower train time per epoch. A normal convolutional layer differs from a depthwise convolution where the depthwise convolution applies the convolution along only one spatial dimension (i.e., channel), whereas a normal convolution applies the convolution across all spatial dimensions or channels at each step. Depthwise separable convolutions are more likely to perform more effectively on deeper models that may have an overfitting problem and on layers with larger kernels because there is a greater decrease in parameters and computations that would offset the high computation cost of performing two convolutions instead of one. Non-linear layers broaden the model’s possibilities, making a deep network superior to a wide network. We use a 1×1 kernel and add an activation layer after it to increase the number of non-linear layers without significantly increasing the number of parameters and computations. This adds a layer of depth to the network. Based on the model structure, our proposed model has two versions: DISubNetV1 and DISubNetV2. Depthwise convolution layers from both subnetworks are concatenated to form the DISubNetV1. Because the depthwise layers are close to the input, it extracts low-level features and concatenates features from both subnetworks to provide more information to the inception module. This version of the model achieves 99.96% accuracy, which is higher than all other models except CNN-Inception. In Figure 16a,b, the accuracy and loss plots of DISubNetV1 exhibit better convergence and fewer fluctuations. The DISubNetV2 concatenates inception modules rather than depthwise layers. At the beginning of the model, the input from different subnetworks goes through different levels of abstraction with different filters. As a result, it enables in obtaining more features when concatenated and provides better classification output. Regarding accuracy, the DISubNetV2 outperformed all other models with a score of 99.98% on thermal data. Although there are a few more fluctuations in the accuracy and loss of DISubNetV2 in Figure 17a,b, there is a better learning curve over the course of training. Even though DISubNetV2 has 0.002 more errors than DISubNetV1, it can still be used as a straightforward model with 4.5 M parameters.

**Figure 18:**
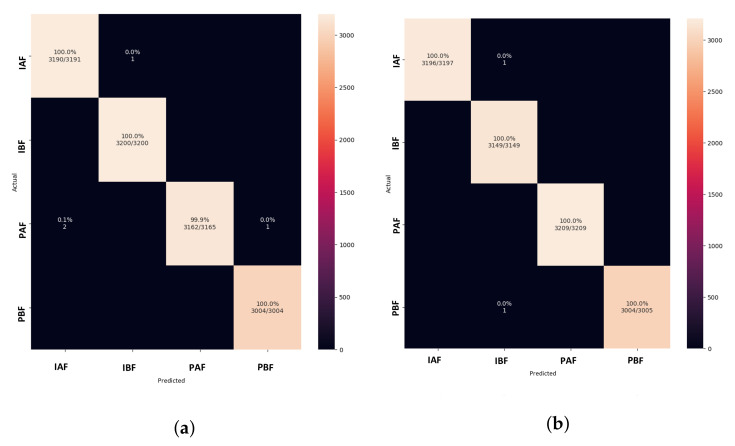
Confusion matrix of proposed DISubNet models for learning rate = 0.001. (**a**) DISubNetV1. (**b**) DISubNetV2.

In comparison with other models, the confusion matrix of both proposed versions in Figure 18a,b shows correctly classified pig treatment classes. As a result, the model outperforms other models trained on thermal data from pig treatments.

Table 1 summarizes our results for learning rate = 0.001. The proposed model DISubNet models, DISubNetV1 and DISubNetV2, provides increased accuracy compared to all the models for pig treatment classifcation.

### 5.2. Comparison with Different Learning Rates

Our proposed models were trained at various learning rates, including $10^{-2}$, $10^{-3}$, and $10^{-4}$. Table 2 summarizes the experiment and includes evaluation metrics such as accuracy, precision, recall, and F1 score.

All models perform better with lower learning rates, such as $10^{-3}$ and $10^{-4}$. Furthermore, for the learning rate of $10^{-4}$, our proposed models outperformed all other models with improved accuracy in the range of 99.96–99.99%. It also clearly shows that at higher learning rates, all models have an accuracy of less than 40% excluding the Xception model. With a learning rate of $10^{-2}$, Xception outperforms all other models with an accuracy of 99.96%. However, the proposed model is smaller in compared to number of parameters. Though VGGNet has a similar accuracy of 99.98% to DISubNetV2, it is a relatively large model with 17.7 M parameters, particularly in comparison to DISubNetv2 which has 4.5 M parameters. The models are unable to converge well when the learning rate is $10^{-2}$, which may be caused by a smaller validation data sample or an uneven distribution of data. Since the dataset for paired before feeding data contains few samples, all models exhibit high learning fluctuations without increasing the accuracy. On the other hand, performance improves when the learning rate is reduced. Therefore, it is obvious that lowering the learning rate when training these models will result in better performance. In a few instances, the unbalanced dataset makes it challenging to learn the model for each batch, producing a high loss value.

### 5.3. Comparison with Number of Parameters and Model Size

In comparison to other models, our proposed models, DISubNetV1 and DISubNetV2, provide few parameters. The number of parameters typically rises when CNN models are expanded, potentially leading to a deeper model. However, this might impact the accuracy gain caused by the vanishing gradient. The depthwise convolution layer model requires fewer parameters and is more accurate. Table 3 compares all models in terms of parameter count and model size (in MB). With 4.5 M parameters, our suggested model yields a size of 53.7 MB.

It is advantageous to have lightweight models in applications that run on mobile devices. Mobile-based deep learning applications have the potential to revolutionize pig farming by providing farmers with real-time data and insights that can help them optimize their operations and improve animal welfare. With the use of mobile-based deep learning applications, farmers can identify each pig in their herd and track their growth and health. This information can be used to monitor individual pig performance and to identify and address any health issues early on. Deep learning models can be trained to analyze pig behavior, such as eating and drinking patterns, activity levels, and social interactions. This information can be used to identify any abnormal behavior, which could be a sign of stress, illness, or other problems. With the use of mobile-based deep learning applications, farmers can use predictive analytics to forecast the growth rate of their pigs, identify potential health problems early, and optimize their feeding and breeding strategies. By monitoring the individual behavior and performance of pigs, farmers can optimize their resource allocation, such as feed and water, and minimize waste. The use of mobile-based deep learning applications can help farmers save time and money by automating data collection and analysis, reducing the need for manual labor, and increasing efficiency.

### 5.4. Importance of Pig Treatment Classification in Animal Welfare

Pig treatment classification can be applied to many aspects of farming and animal care. The goal of the model is to create a framework for a decision support system for predictive analytics that can be used to identify changes in pig behaviour in response to environmental perturbations such as shifts in playtime, feeding interval time, and rest time. Isolated pigs develop behavioral stress reactions. Pigs that are completely isolated continue to display behavioural signs of stress, whereas pigs that are partially isolated (contact through a fence) eventually display fewer behavioral signs of stress [58]. Researchers working with animals can use these data to advocate for better treatment of animals. Future monitoring and treatment could benefit from using a non-invasive thermal camera to record the skin’s surface temperature. In veterinary medicine, thermal imaging is used to help diagnose diseases and to detect (early) signs of pain or stress in animals. Thermal imaging can also detect postoperative inflammation and changes in blood flow to the surgical site. Therefore, thermal images are a useful tool for identifying issues that may impact animal welfare.

## 6. Conclusions

This paper proposed the DISubNetV1 and DISubNetV2 models, which are made up of depthwise convolution layers and inception modules for classifying pig treatments. Various evaluation metrics are used to compare the proposed model to LeNet5, AlexNet, VGGNet, Xception, CNN-LeakyReLU, and CNN-inception models. The versions differ in terms of the concatenation of the layers in the subnetworks. Based on thermal data, the models classify four pig treatment categories. The proposed model outperforms all other models with fewer parameters and higher accuracy. Although the model improves accuracy, it misclassifies one of the paired before-feeding classes. It also shows fluctuations in learning due to the uneven distribution of the data. In the future, we plan to use this research for other applications such as emotion recognition to provide better information based on the features learned in the pig treatment classification. Since only thermal images were used, we intend to use videos instead. In addition, the conversion of thermal scores to grayscale may have resulted in the loss of some features. Therefore, future work on the model must target the feature loss to improve its accuracy.

## Figures and Tables

**Table 1:** Performance comparison of different models with learning rate = 0.001.

Model	Accuracy	Precision	Recall	F1 Score	Loss
	(%)	(%)	(%)	(%)	
LeNet5	99.9045	99.9045	99.9045	99.9045	0.0061
AlexNet	90.2229	90.2581	90.2581	90.2229	0.2716
VGGNet	85.4379	86.3148	85.5091	85.4379	0.4164
Xception	99.9522	99.9522	99.9522	99.9522	0.0043
CNN-LeakyReLU	99.1401	99.1426	99.1401	99.1403	0.0976
CNN-inception	99.9761	99.9761	99.9761	99.9761	0.0179
DISubNetV1	99.9682	99.9682	99.9682	99.9682	0.0014
DISubNetV2	99.9841	99.9841	99.9841	99.9841	0.0036

**Table 2:** Comparison of all models with different learning rates.

Model	Learning	Accuracy	Precision	Recall	F1 Score
	Rate	(%)	(%)	(%)	(%)
LeNet5	0.01	25.3025	6.4022	25.3025	10.2188
	0.0001	99.9124	99.9125	99.9124	99.9124
AlexNet	0.01	24.8885	6.1944	24.8885	9.9199
	0.0001	99.9602	99.9602	99.9602	99.9602
VGGNet	0.01	25.6528	6.5806	25.6528	10.4744
	0.0001	99.9840	99.9840	99.9840	99.9840
Xception	0.01	99.9682	99.9682	99.9682	99.9682
	0.0001	99.9920	99.9920	99.9920	99.9920
CNN-LeakyReLU	0.01	25.8996	6.7079	25.8996	10.6560
	0.0001	99.9601	99.9601	99.9601	99.9601
CNN-inception	0.01	37.7388	50.2383	37.7388	32.1467
	0.0001	99.9681	99.9681	99.9681	99.9681
DISubNetV1	0.01	25.1433	6.3218	25.1433	10.1034
	0.0001	99.9682	99.9682	99.9682	99.9682
DISubNetV2	0.01	25.2229	6.3619	25.2229	10.1610
	0.0001	99.9920	99.9920	99.9920	99.9920

**Table 3:** Model parameters and size comparison.

Model	Number of Parameters	Model Size
LeNet5	19,628,074	224 MB
AlexNet	23,392,580	267 MB
VGGNet	17,075,396	195 MB
Xception	20,991,980	240 MB
CNN-LeakyReLU	7,255,332	83.2 MB
CNN-inception	7,419,812	85.8 MB
DISubNetV1	4,591,574	53.7 MB
DISubNetV2	4,591,574	53.7 MB

## Data Availability

The data that support the findings of this study are available from the authors upon reasonable request.
